# Igm-enriched polyclonal immunoglobulins in an experimental model of gram negative pneumonia developing septic shock

**DOI:** 10.1186/2197-425X-3-S1-A795

**Published:** 2015-10-01

**Authors:** R Vaschetto, N Clemente, A Pagni, T Esposito, F Mercalli, R Boldorini, F Della Corte, A Chiocchetti, U Dianzani, P Navalesi

**Affiliations:** Ospedale Maggiore della Carità, Anestesia e Rianimazione, Novara, Italy; Università del Piemonte Orientale 'Amedeo Avogadro', Dipartimento di Scienze della Salute, Novara, Italy; Università del Piemonte Orientale 'Amedeo Avogadro', Dipartimento di Medicina Traslazionale, Novara, Italy; Università del Piemonte Orientale 'Amedeo Avogadro', Anatomia Patologica, Novara, Italy

## Introduction

Severe pneumonia is a major challenge in the Intensive Care Unit, as it is characterized by high morbidity and mortality. A high rate of patients with pneumonia, in particular ventilator-associated poneumonia, develops septic shock. Although some interesting results have been reported in uncontrolled studies where IgM-enriched human intravenous immunoglobulins were added to the standard treatment of septic shock, a well-conducted clinical trial is missing[1]. Also, physiopathological data supporting such a trial are presently insufficient.

## Objectives

We aimed to evaluate if Pentaglobin, a commercially available IgM-enriched polyclonal preparation, reduced pulmonary and systemic inflammation in an experimental model of Gram negative pneumonia causing septic shock. Secondary endpoints were the assessment of the effects on respiratory and hemodynamic stability and survival.

## Methods

The protocol was approved by the University Ethic Committee. Thirty-eight Sprague Dawley rats were ventilated with a mildly injurious ventilation i.e., pick pressure of 25 cmH2O and positive end-expiratory pressure of 5 cmH2O for 30 minutes to prime the lung. The rats were subsequently randomized to received intratracheal instillation of either LPS (12 mg/kg) or placebo followed by 3.5 hours of protective mechanical ventilation. Pentaglobin at 25 mg/h (0.5 ml/h) or saline were double-blindly intravenously administered in the last hour of mechanical ventilation. During the experiment, gas exchange and hemodynamic measurements were recorded. Thereafter, the animals were sacrificed, and blood and organs were stored for cytokines measurements.

## Results

In the bronchoalveolar lavage of the LPS-treated rats, TNF-alpha levels resulted significantly lower in the Pentaglobin group compared to placebo (5 [5-755] ng/ml vs. 1406 [971-2271] ng/ml), p < 0.05. Pentaglobin significantly increased the plasmatic level of IL-10 compared to placebo (389 [121-522] pg/ml vs. 238 [82-259] pg/ml), p < 0.05. No differences were found in respiratory and hemodynamic variables. All the control animals survived for 4 hours. For the rats challenged with LPS, survival was significantly reduced in the placebo group i.e., 63%, compared to controls, p = 0.03, while no differences were found between those treated with Pentaglobin (83% survival) and those of the control group.

## Conclusions

In a pneumonia model causing septic shock, the administration of Pentaglobin as rescue treatment reduced the inflammatory response in lung and blood, although no difference was found in respiratory and hemodynamic variables. Survival was significantly reduced in LPS-placebo treated animals compared to controls but not in LPS group treated with Pentaglobin.

## Grant Acknowledgment

Pentaglobin was kindly donated by Biotest.
